# Update on Chemotherapeutic Approaches and Management of Bevacizumab Usage for Glioblastoma

**DOI:** 10.3390/ph13120470

**Published:** 2020-12-16

**Authors:** Yusuke Funakoshi, Nobuhiro Hata, Daisuke Kuga, Ryusuke Hatae, Yuhei Sangatsuda, Yutaka Fujioka, Kosuke Takigawa, Masahiro Mizoguchi

**Affiliations:** Department of Neurosurgery, Graduate School of Medical Sciences, Kyushu University 3-1-1 Maidashi, Higashi-Ku, Fukuoka 812-8582, Japan; sf1wan0610@gmail.com (Y.F.); kuga@ns.med.kyushu-u.ac.jp (D.K.); ryhatae@ns.med.kyushu-u.ac.jp (R.H.); y-sangat@med.kyushu-u.ac.jp (Y.S.); yfujioka@med.kyushu-u.ac.jp (Y.F.); taki1221@med.kyushu-u.ac.jp (K.T.); mmizoguc@ns.med.kyushu-u.ac.jp (M.M.)

**Keywords:** bevacizumab, glioblastoma, vascular endothelial growth factor (VEGF), chemotherapy, survival

## Abstract

Glioblastoma, the most common primary brain tumor in adults, has one of the most dismal prognoses in cancer. In 2009, bevacizumab was approved for recurrent glioblastoma in the USA. To evaluate the clinical impact of bevacizumab as a first-line drug for glioblastoma, two randomized clinical trials, AVAglio and RTOG 0825, were performed. Bevacizumab was found to improve progression-free survival (PFS) and was reported to be beneficial for maintaining patient performance status as an initial treatment. These outcomes led to bevacizumab approval in Japan in 2013 as an insurance-covered first-line drug for glioblastoma concurrently with its second-line application. However, prolongation of overall survival was not evinced in these clinical trials; hence, the clinical benefit of bevacizumab for newly diagnosed glioblastomas remains controversial. A recent meta-analysis of randomized controlled trials of bevacizumab combined with temozolomide in recurrent glioblastoma also showed an effect only on PFS, and the benefit of bevacizumab even for recurrent glioblastoma is controversial. Here, we discuss the clinical impact of bevacizumab for glioblastoma treatment by reviewing previous clinical trials and real-world evidence by focusing on Japanese experiences. Moreover, the efficacy and safety of bevacizumab are summarized, and we provide suggestions for updating the approaches and management of bevacizumab.

## 1. Introduction

Glioblastoma (GBM) is the most common primary brain tumor in adults and is known to have one of the most dismal prognoses in cancer. The conventional standard treatment for newly diagnosed GBM was established in 2005 when a multimodal treatment consisting of maximal safe resection, concomitant temozolomide (TMZ) and radiotherapy, followed by adjuvant TMZ showed improvements in GBM prognosis [1]; the resulting median overall survival (OS) was reported to be only approximately 14–16 months [2,3,4]. The Food and Drug Administration (FDA) has also approved the use of a tumor treating fields (TTF) device for treatment of recurrent GBM in 2011 and newly diagnosed GBM in 2015 [5]. The addition of TTF device treatment to the conventional TMZ standard therapy improved median OS by 20.9 months [5,6]. However, despite aggressive and advanced treatment strategies, the prognosis of patients with GBM remains poor. Notably, multidisciplinary treatment options for GBM are limited compared with other carcinomas; therefore, there is a significant need for identifying and assessing putative treatment options for these patients.

Neo-angiogenesis in GBM suggests a possible utility for anti-angiogenic therapies. Malignant gliomas are known to have a hypervascular nature and high expression levels of vascular endothelial growth factor (VEGF) [7]. Angiogenesis in brain tumor biology is important because endothelial proliferation is a hallmark of GBM and is considered a major criterion for histopathological diagnosis [8]. In addition, GBM cells produce angiogenic factors and signaling pathways, resulting in new vessel formation that supports continued tumor growth [9,10,11].

Bevacizumab (BEV) is a molecular-targeted drug that inhibits angiogenesis by interrupting the VEGF/VEGF-receptor signaling pathway and leads to indirect antitumor activity [12]. BEV has been approved for multiple cancers including colon, lung, kidney, and cervix cancers by the FDA in 2003, 2006, 2009, and 2014, respectively [13]. In 2009, the FDA approved BEV for recurrent GBM based on the success of two phase II clinical trials, AVF3708g and National Cancer Institute 06-C-0064E [14,15]. Thereafter, BEV acquired world-wide recognition as a second or subsequent line option for the treatment of gliomas due to the corticosteroid-sparing effect and, in part, because a proportion of patients show long-term responses [16]. However, the survival benefit of BEV for patients with GBM remains controversial due to a lack of clear evidence.

To evaluate the clinical impact of BEV as a first-line drug for GBM, two randomized clinical trials, AVAglio and RTOG 0825, were performed. These clinical trials indicated that BEV as an initial treatment improves the progression-free survival (PFS) of patients with GBM [17,18] and is beneficial for maintaining patient performance status (PS) [19]. These outcomes led to BEV approval in Japan in 2013 as an insurance-covered first-line drug for GBM concurrently with its second-line application. However, prolongation of overall survival (OS) was not evinced in these clinical trials; hence, the clinical benefit of BEV for newly diagnosed GBMs remains controversial. In addition, a recent meta-analysis of randomized controlled trials of BEV combined with TMZ in recurrent GBM showed an effect only on PFS [20] and that the benefit of BEV even for recurrent GBM is controversial. In this review, we discuss the clinical impact of BEV for the treatment of GBM by reviewing previous clinical trials and real-world evidence by focusing on Japanese experiences. Moreover, the efficacy and safety of BEV are summarized, and we provide suggestions for updating the approaches and management of BEV.

## 2. First-Line BEV

The two randomized phase III clinical trials AVAglio and RTOG 0825 failed to prove the impact of BEV on the OS of patients with newly diagnosed GBM [17,18]; thus, first-line BEV has not been globally accepted as an evidence-based regimen and clinical studies have been limited since these two clinical trials. Notably, AVAglio indicated that BEV improves only the PFS of patients with GBM [17]; however, an exploratory AVAglio study reported prolonged OS with first-line BEV in patients who did not undergo second-line treatments [21]. This suggests that a favorable impact of BEV can be observed when clinical trials are designed to evaluate its efficacy for patients with severe conditions.

Table 1 summarizes the outcomes of recent clinical studies of first-line BEV conducted after AVAglio and RTOG 0825. The GENOM 009 randomized phase II trial demonstrated the positive impact of BEV in patients with newly diagnosed GBM; the PFS and OS of unresected patients with newly diagnosed GBM tended to be longer in the TMZ + BEV group than in the TMZ alone group [TMZ + BEV vs. TMZ median PFS: 4.8 vs. 2.2 months, HR 0.70 (0.46–1.07), *p* = 0.10; median OS: 10.6 vs. 7.7 months, HR 0.68 (0.44–1.04), *p* = 0.07] [22]. The combination of BEV + TMZ is reported to be more active than TMZ alone and may confer benefits in terms of tumor shrinkage in unresected patients [22]. Moreover, BEV is expected to provide a potential benefit for elderly patients with GBM because BEV may wean off steroids and reduce symptomatic radiation necrosis [17,23]. The efficacy of BEV for elderly patients with newly diagnosed GBM was evaluated in some clinical trials. For instance, the ATAG phase II trial reported that TMZ + BEV is active in elderly patients with GBM with low PS and had an acceptable tolerance level [24]; however, TMZ + BEV was not compared to TMZ alone in this study. In the ARTE randomized phase II trial, hypofractionated radiotherapy (40 Gy in 15 fractions) with BEV or without BEV in patients with newly diagnosed GBM aged ≥ 65 years was assessed, and although PFS was longer in the BEV group than in the group without BEV, OS was similar (BEV vs. without BEV median PFS: 7.6 vs. 4.8 months, *p* = 0.003; median OS: 12.1 vs. 12.2 months, *p* = 0.77) [25]; thus, the outcomes of ARTE do not support the hypothesis that the addition of BEV to radiotherapy prolongs survival of elderly patients with GBM. Furthermore, BEV in combination with cytotoxic agents other than TMZ for patients with newly diagnosed GBM was evaluated. The GLARIUS randomized phase II trial explored BEV + irinotecan as an alternative to TMZ for patients with newly diagnosed GBM, unmethylated O^6^-methylguanine-DNA methyltransferase (*MGMT*) status, and for whom the TMZ regimen is expected to be less beneficial [26]. As a result, PFS improved in the BEV + irinotecan group compared with the TMZ group but there was no difference in OS [BEV + irinotecan vs. TMZ median PFS: 9.7 vs. 6.0 months, HR 0.57 (0.41–0.80), *p* < 0.001; median OS: 16.6 vs. 17.5 months, HR 1.02 (0.71–1.48), *p* = 0.55] [26]. Overall, the results indicate that, although BEV may have some clinical benefit for severe clinical conditions, such as unresectable tumors or advanced age, there is no sufficient evidence in these clinical trials.

Few clinical trials can provide sufficient evidence of the benefit of BEV for newly diagnosed GBM because a favorable impact of BEV can be demonstrated in patients with more severe conditions that tend to be obstructive for enrolment in randomized trials. Accordingly, real-world data from retrospective studies conducted by Japanese institutions appear promising in providing evidence of first-line BEV efficacy for patients with severe clinical conditions. Yonezawa et al. [27] retrospectively analyzed newly diagnosed malignant glioma and reported that the use of BEV is an independent beneficial factor associated with prolonged OS in patients with unresectable GBM [BEV vs. non-BEV median OS: 566 vs. 160 days, HR 0.25 (0.10–0.65), *p* = 0.001]. We previously demonstrated that first-line BEV prevents deterioration during concurrent TMZ treatment and radiotherapy, and contributes to OS prolongation in patients with unresectable tumors (BEV vs. without BEV median OS: 17.4 vs. 9.8 months, *p* = 0.075) [28]; this highlights the advantages of first-line BEV for severe clinical conditions such as unresectable tumors and poor PS (*p* = 0.017). In addition, in our cohort, poor prognostic patients with GBM, unmethylated *MGMT* status, treated with BEV + TMZ showed improved OS (TMZ + BEV vs. TMZ median OS: 16.7 vs. 12.2 months, *p* = 0.04) [29].

The paradoxical PFS and OS results of randomized phase III clinical trials [17,18] suggest that early BEV use can accelerate clinical course after progression. A possible reason for the failure of BEV to improve OS despite its prolongation of PFS in clinical trials is that BEV treatment may show changes in imaging findings that are not necessarily correlated with beneficial effects on tumor biology [30]. However, recent real-world data from Japanese institutes, in which BEV is available as a first-line treatment, indicate improved survival with selective administration of first-line BEV combined with TMZ for patients with severe clinical conditions such as unresectable cases or poor PS [27,28,29,31]. Nevertheless, the evidence level is not sufficiently high as these reports are institutional and retrospective studies, and BEV usage is institution-specific. Moreover, the FDA has approved BEV use only for recurrent GBM, whereas first-line BEV concurrent with its second-line application has been approved only in Japan. The unique BEV approval in Japan, regardless of clinical stage, has had a positive impact on the survival of specific patients. Further accumulation of evidence in addition to clinical data, such as from Japanese cohorts, is warranted to evaluate the real-world impact of BEV therapy.

## 3. Second or Subsequent Line BEV

Various phase II clinical trials with BEV have shown promising results in terms of PFS of recurrent GBM [15,32,33]. Since its approval by the FDA in 2009, BEV efficacy for recurrent GBM has been reported in numerous studies, but further studies have not found any improvement in OS or a relatively high response rate. Several clinical trials were initiated to explore the efficacy of combination therapy with BEV, and although such therapy improves PFS significantly, there is no sufficient evidence of OS prolongation [20]. A recent meta-analysis of randomized controlled trials of BEV + TMZ for recurrent GBM also showed an effect only on PFS [20]. Although OS prolongation was not evinced and the clinical benefit of BEV for GBM remains controversial, BEV continues to be used in some settings, especially for recurrent GBMs that have failed standard therapies or do not qualify for targeted therapies.

Recent studies have reported on the usage of BEV, such as the dose, timing, and combined treatment options for patients with GBM. BEV prevents the growth of new blood vessels, resulting in suppressed tumor growth. Because this anti-tumor effect only has an indirect impact on GBM growth, BEV in combination with cytotoxic agents, which have a direct impact, has been well studied. However, no studies have provided sufficient evidence supporting the efficacy of combination therapy with these agents. Over the past 10 years, several phase II studies have investigated BEV in combination with various agents, including irinotecan, cetuximab, carboplatin, erlotinib, sorafenib, and dasatinib [34,35,36,37,38,39,40,41]. Representing a positive report on the effect of combined therapy with BEV, the randomized phase II BELOB trial in 2014 demonstrated a potential benefit of BEV when combined with lomustine for patients with recurrent GBM (BEV + lomustine vs. BEV vs. lomustine median PFS: 4 vs. 3 vs. 1 month; median OS: 12 vs. 8 vs. 8 months) [42]. Using tumor material from participants in the BELOB trial, Erdem-Eraslan et al. [43] reported that tumors assigned to IGS-18 or classical GBM and treated with BEV + lomustine showed a significant benefit in PFS and a trend toward improved OS (BEV + lomustine vs. BEV vs. lomustine median PFS: 4.2 vs. 2.9 vs. 1.4 months; median OS: 11.9 vs. 8.3 vs. 7.9 months]). Table 2 summarizes the outcomes of recent clinical studies since the BELOB trial. To validate the efficacy of BEV + lomstine, Wick et al. [44] conducted a phase III clinical trial; however, it failed to confirm a survival benefit [BEV + lomstine vs. lomstine median PFS: 4.2 vs. 1.5 months, HR 0.49 (0.39–0.61), *p* < 0.001; median OS: 9.1 vs. 8.6 months, HR 0.95 (0.74–1.21), *p* = 0.65]. Moreover, the efficacy of BEV + lomstine for recurrent GBM after first-line BEV + TMZ was evaluated in a phase II clinical trial, but no survival benefit was observed [BEV + lomstine vs. lomstine median PFS: 2.3 vs. 1.8 months, HR 0.70 (0.37–1.33); median OS: 6.4 vs. 5.5 months, HR 1.04 (0.69–1.59)] [45]. Field et al. [46] also compared BEV + carboplatin with BEV alone as a second-line therapy in a phase II clinical trial; however, adding carboplatin resulted in more toxicity without additional clinical benefit (BEV + carboplatin vs. BEV median PFS: 3.5 vs. 3.5 months, HR 0.92 (0.64–1.33), *p* = 0.66; median OS: 6.9 vs. 7.5 months, HR 1.18 [0.82–1.69], *p* = 0.38).

Several clinical trials have evaluated the optimal dosage of BEV. Weathers et al. [47] compared low-dose BEV (5 mg/kg) + lomustine with standard-dose BEV (10 mg/kg) and found a trend toward improved PFS in the low-dose BEV + lomustine group; however, no significant difference in OS was observed [low-dose BEV + lomustine vs. standard dose BEV median PFS: 5.0 vs. 3.2 months, *p* = 0.08; median OS: 13.1 vs. 8.8 months, *p* = 0.98]. Ajlan et al. [49] also reported in their retrospective study that a lower dose of BEV (< 3 mg/kg/week) may be more efficacious. In their cohort, including 9% first-line BEV and 91% second or subsequent line BEV, severe adverse events did not occur and the median OS was 39.0 months [49]. Although a 5 mg/kg/week dose is routinely used for recurrent glioma, we may need to review the appropriate dose of BEV administration. Additionally, Badruddoja et al. [48] reported that biweekly TMZ + BEV (intravenous, 10 mg/kg) on days 1 and 15 of a 28-day cycle combined with TMZ (100 mg/m^2^) on days 1–5 and 15–19 of a 28-day cycle was well tolerated and may be a salvage regimen for patients with recurrent GBM.

Franceschi et al. [50] reported that BEV as a third-line drug in patients with GBM may be feasible and well-tolerated. Although it was a retrospective study and their cohort was relatively small, including 23 patients in the BEV alone group, 5 in BEV + irinotecan, and 6 in BEV + lomustine, both PFS and OS starting from the initiation of third-line BEV groups were significantly longer than those treated with other chemotherapies (BEV vs. other chemotherapy median PFS: 4.7 vs. 2.6 months, *p* = 0.020; median OS: 8.0 vs. 6.0 months, *p* = 0.014) [50]. Thus, BEV may also be indicated for third-line therapy of GBM.

Thus, although the benefit of BEV on OS of patients with recurrent GBM has not been proven, combination therapy with BEV improves PFS of patients with recurrent GBM, and monotherapy with BEV may have benefits in some settings, e.g., as a third-line therapy for GBM. In addition to evaluating first-line BEV, further efforts to address the appropriate usage of BEV for patients with recurrent GBM, including dose, timing, and combined agents, are required to maximize the clinical benefits of BEV.

## 4. Population-Based Analysis

Recently, population-based analyses were conducted to investigate changes in the survival of patients with GBM. For example, a 2018 study compared OS between patients diagnosed with GBM before and after BEV approval using US population-based cancer registry data (SEER) [51]; this study included 6120 patients in the pre-BEV cohort and 6753 patients in the post-BEV cohort, and the adjusted hazard of death was found to be significantly lower in the post-BEV cohort. Zhu et al. [52] also compared three large groups: pre-radiotherapy-TMZ and pre-BEV (*n* = 9526), post-radiotherapy-TMZ and pre-BEV (*n* = 8706), and post-radiotherapy-TMZ and post-BEV (*n* = 10,701), and concluded that the OS of patients with GBM has steadily improved, likely resulting from the administration of TMZ concomitant with radiotherapy for newly diagnosed GBM and then BEV for recurrent GBM after its FDA approval.

In Japan, similar investigations are currently underway. In 2002, the Japanese government introduced a per diem prospective payment system with a diagnosis-related group-like grouping, termed Diagnostic Procedure Combination (DPC) [53]. Using the DPC database, we have previously reported on the current trends and healthcare resource usage in the treatment of primary malignant brain tumors in Japan (J-ASPECT study-Brain Tumor) [54]. In addition, the Japan Neurosurgical Society established the Japan Neurosurgical Database, a nationwide, hospital-based, multi-center registry in 2016; thus, analysis of real-world clinical practice in Japan is expected to develop further in the near future [55]. Because first-line BEV is approved only in Japan, and Japanese BEV usage is unique, the population-based analysis of Japanese real-world data may provide indirect evidence validating the efficacy of BEV treatment. We are currently analyzing the DPC database and planning to report the current trend in GBM treatment after BEV approval in Japan. Population-based analysis of further real-world data can accumulate sufficient evidence to support BEV usage.

## 5. Evaluation of BEV Response

The evaluation of GBM recurrence is complicated by treatment-induced changes and discordance between enhancing and non-enhancing magnetic resonance imaging (MRI) [56]. To evaluate GBM relapse, we should consider pseudoprogression and pseudoresponse. Pseudoprogression has been reported in up to 31% of patients with gliomas treated with radiotherapy + TMZ [57,58,59,60]. In pseudoprogression, radiotherapy + TMZ increases the blood–brain barrier permeability causing leakage of contrast media in the brain, producing a large area of enhancement during MRI, which mimics tumor growth [61]. In contrast, BEV normalizes blood –brain barrier permeability and blood flow shortly after treatment initiation, which decreases the enhancement without a real antitumor effect, leading to a pseudoresponse [62]. Pseudoprogression and pseudoresponse most likely to occur during the first 3 months of treatment [56]. In 2010, the Response Assessment in Neuro-Oncology (RANO) working group recommended that the assessment of non-contrast-enhancing tumor components should be considered as a response criterion [63].

Numerous studies have reported atypical patterns of progression after BEV treatment, such as multifocal and widely disseminated disease, and there are concerns that BEV increases the risk of concomitantly eliciting tumor adaptation and progression to stages with greater malignancy and heightened invasiveness [64]. After administration of BEV, the receptor tyrosine kinase c-Met was found upregulated in GBM cells, which is considered to promote tumor hypoxia and invasion, leading to tumor growth and therapeutic resistance [65]. However, a significant difference in the relapse pattern was not observed in the AVAglio study [17], and the impact of BEV on relapse pattern is controversial.

Table 3 summarizes the relapse pattern and prognosis of GBM patients treated with BEV. Iwamoto et al. [66] reported that increased non-enhancing tumor with the decreased or stable enhancing disease is a common pattern of GBM progression during BEV therapy. The authors classified relapse patterns into three groups according to (1) enhancing initial site, (2) new enhancing, and (3) non-enhancing tumor, and demonstrated that non-enhancing tumors after BEV treatment are correlated with worse survival (HR 2.25 (1.00–5.10), *p* = 0.05). However, Pope et al. [67] scored tumor patterns as local, distant, diffuse, or multifocal, and demonstrated that survival was similar for patients with different patterns of recurrence [local to diffuse vs. local to local, BEV group median OS: 9.2 vs. 9.7 months, HR 1.6 (0.78–3.17); BEV + irinotecan median OS: 8.9 vs. 11.5 months, HR 1.6 (0.83–3.16)]. As another representative classification, Nowosielski et al. [68] categorized the relapse patterns after BEV treatment according to (1) exclusively T2-diffuse hyperintense tumor progression without new or only speckled contrast enhancement (CE); (2) initial decrease in and a subsequent flare-up of CE at progression; (3) no decrease in CE or development of new lesions at first follow-up imaging; and (4) exclusively T2-circumscribed hyperintense tumor progression without new or only speckled CE. The authors reported that patients with T2-diffuse hyperintense tumor progression and flare-up of CE survived longer than primary non-responders or patients with T2-circumscribed hyperintense tumor progression (T2 diffuse vs. flare-up of CE vs. primary non-responder vs. T2-circumscribed hyperintense tumor median OS: 4.8 vs. 4.6 vs. 3.0 vs. 1.6 months) [68]. Kim et al. [69] adopted a similar classification and demonstrated that non-enhancing infiltration after BEV treatment is not associated with worse prognosis (primary non-responder vs. flare-up of CE vs. non-enhancing infiltration median OS: 4.4 vs. 11.0 vs. 9.0) and that discontinuation of therapy can aggravate the clinical course.

Although atypical patterns of progression after BEV treatment, such as multifocal and widely disseminated disease, have been noted, Chamberlain [71] reported that non-local diseases, such as diffuse, distant, or multifocal, do not appear to impact survival or increase in incidence after BEV treatment. Bloch et al. [70] also reported that the risk of dissemination is not considerably increased as a result of BEV use and that the progression pattern does not affect subsequent survival. Mamo et al. [72] compared the progression pattern of first-line BEV with that of second-line or later, and found no difference in progression patterns between these groups.

In our cohort, atypical relapse patterns, including non-enhancing tumor or diffuse progression, increased after BEV approval, but they did not significantly influence the survival of patients with GBM, as was the case in other studies. Taken these findings into consideration, the hypotheses that BEV accelerates aggressive disseminated progression and that non-enhancing tumors are correlated with worse survival are not consistent.

## 6. Predictive Markers of BEV

AVAglio subanalysis indicated that the benefit of BEV on OS occurred only in patients with newly diagnosed GBM who had pro-neural *IDH*-wildtype tumors, which was associated with poorer prognosis in the cohort [73]. For some newly diagnosed GBM, BEV may have clinical benefits. Although predictive markers of BEV have been reported, there is no sufficient evidence. To confirm the reproducibility of these markers, further real-world data need to be accumulated and validated.

### 6.1. Genetic Markers

Both AVAglio and RTOG 0825 included an optional biomarker component. Preliminary analysis in the AVAglio study did not identify a predictive value for baseline plasma VEGF-A or VEGF receptor-2 [74]. RTOG 0825 subanalysis, however, identified a 10-gene signature profile that was predictive of both PFS and OS among patients receiving BEV [75]. *EGFR* amplification and the classical subtype were reported to be associated with poor response to BEV [76]. In addition, we previously reported that patients with unmethylated *MGMT* status have a high therapeutic sensitivity to BEV [29], and in our article under review, we demonstrate the positive impact of BEV, particularly in patients with unmethylated *MGMT* status harboring *CDKN2A* homozygous deletions.

### 6.2. Imaging Markers

In addition to these genetic markers, some imaging markers have been recently reported. Representative imaging markers include enhancing tumor volume measurements, relative cerebral blood volume variation, perfusion maps, and contrast-enhanced T1-weighted subtraction maps [77,78,79]. Recently, Grossmann et al. [80] reported that radiomics provide prognostic value for survival and progression of patients with GBM receiving BEV treatment. In addition, Lee et al. [81] demonstrated that diffusional kurtosis imaging, which is an extension of diffuse tensor imaging, allows the detection of tissue changes 28 days after initiating BEV treatment and may provide information regarding tumor progression.

### 6.3. Other Markers

A recent study reported that basal neutrophils and regular T cells in peripheral venous blood are significantly associated with survival during treatment with BEV [16].

## 7. Advantages of BEV

There are several potential therapeutic advantages of anti-angiogenic therapies, including BEV, in the treatment of GBM. Previous clinical data suggest that patients whose tumor perfusion or oxygenation increases in response to anti-angiogenic therapies may actually survive longer; hence, strategies aimed at alleviating tumor hypoxia while improving perfusion may enhance the outcome of radiotherapy, chemotherapy, and immunotherapy [82].

### 7.1. Tumor and Radiation Sensitivity

Because the newly formed vasculature of GBM has a selective vulnerability, anti-angiogenic therapies show tumor selectivity and there is less concern about drug delivery [8]. Regarding BEV in combination with radiotherapy, a previous in vivo study suggested synergistic effects of anti-angiogenic therapies in combination with radiotherapy; in experimental animals, anti-angiogenic therapies normalize tumor vasculature and increase tumor oxygenation with a possible improvement in radiosensitivity [83]. After BEV treatment of GBM, a decline in the expression of hypoxia-inducible factor (HIF)-1α, an endogenous marker for tumor hypoxia, was reported [84]. In addition, ^18^F-fluoromisonidazole (FMISO) was able to evaluate the dynamic biological effects of tissue hypoxia and vascular normalization occurring within GBMs treated with BEV [85]. These reports suggest that BEV increases tumor oxygenation and improves radiosensitivity. The efficacy of re-irradiation combined with BEV for the recurrence of high-grade gliomas has also been reported, and more aggressive radiotherapy compared with conventional treatment is expected to be performed [86].

### 7.2. Combination with Immunotherapy

BEV is known to enhance the effects of immunotherapy because VEGF-A suppresses antitumor immunity by inhibiting the maturation of dendritic cells and stimulating the proliferation of regulatory T cells [87]. Indeed, the combination of atezolizumab, an anti-programmed cell death ligand-1 (PD-L1) antibody and BEV induces a strong and synergistic antitumor effect on tumors with high levels of VEGF-A via the induction of cytotoxic T lymphocytes [88]. Tamura et al. [84] demonstrated that BEV downregulates the expression of programmed cell death-1 (PD-1) and PD-L1 immune checkpoint molecules and decreases the number of immunosuppressive regulatory T cells and tumor-associated macrophages.

### 7.3. Effect of Radiation Necrosis

It is well known that BEV can prevent symptomatic radiation necrosis through decreased vascular permeability [89,90], which can lead to the optimization of alternative aggressive radiotherapy regimens. However, in a phase II trial where hypo-intensity modulated radiation therapy (IMRT) (60 Gy in 10 fractions) in combination with TMZ + BEV for newly diagnosed GBM was applied, the rate of presumed radiation necrosis was much higher than anticipated [91]. Further clinical studies are necessary to determine the extent to which aggressive radiotherapy in combination with BEV is allowed.

### 7.4. Anti-Oedematous Effect and Maintenance of PS

Blocking the VEGF pathway restores tumor vasculature to a more normal state, reducing vascular permeability and the regional cerebral blood volume around the tumor [92]. This mechanism decreases oedema around the tumor, and 30–70% of patients who received BEV treatment were reported to have reduced their steroid doses [18,93]. Gramatzki et al. [94] conducted an epidemiological study including 310 patients with GBM in the Canton of Zurich, Switzerland, and reported that the baseline dose of corticosteroids was reduced by more than half in 83% of patients on BEV compared with 48% of patients treated with BEV-free regimens.

The anti-oedematous effect of BEV may also be associated with the maintenance of PS in patients with GBM [95,96]. Patients with GBM show neurological disorders and have low PS even at initial presentation. PS in more than 50% of patients is reported to be low at the primary diagnostic stage [97]. In the AVAglio trial, the results revealed a positive effect on health-related quality of life from adding BEV to standard radiation and TMZ chemotherapy regimens for patients with GBM [98]. Moreover, in our cohort, a favorable outcome of additional BEV was achieved for patients with GBM, especially those with a poor PS, suggesting that additional BEV may contribute to the prevention of early clinical deterioration [28].

## 8. Adverse Events and Management of BEV

### 8.1. Adverse Events

Common and significant toxicities of BEV include hypertension, proteinuria, risk of renal failure, posterior leukoencephalopathy syndrome, venous and arterial thromboembolic disease, bowel perforation, and poor wound healing [8]. In our cohort, deep venous thrombosis was the only apparent adverse event that resulted in the discontinuation of BEV treatment [28]. In addition to these well-recognized adverse events, a previous study reported that prolonged BEV treatment is associated with brain atrophy [99]. Although the relationship between BEV and brain atrophy is controversial, in vitro, long-term BEV treatment was found associated with a reduction in the dendritic length of hippocampal neurons [100].

### 8.2. Management

Because data on the occurrence and optimal management of these treatment-related complications in patients with GBM are limited, management is based on data collected from the experiences of patients with a variety of systemic cancers. Therefore, we need to establish appropriate BEV management for patients with GBM in the future. Although a previous report suggested that discontinuation of BEV treatment can lead to a rebound effect [101], another study demonstrated that the treatment interval of BEV is not associated with a poor outcome [102]. Anderson et al. [103] reported that discontinuation of BEV does not cause rebound recurrence or worsen prognosis but rather improves the response to salvage therapy. At our institute, BEV treatment was generally performed according to the AVAglio regimen [17]; however, tapering or discontinuation of BEV occurred after approximately 6 months as per the physician’s decision based on the evaluation of improvements in clinical conditions and/or radiological findings. Considering the potential risk of continuous BEV leading to deterioration due to adverse events, per protocol use of BEV until progression occurs seems to be less beneficial, especially for patients with a long clinical course. We speculate that our adaptable treatment strategy, first-line BEV optimized for patients with severe clinical conditions and allowing BEV tapering/discontinuation depending on clinical status, maximizes the advantages of the unique BEV approval in Japan and leads to favorable outcomes that were not achieved in clinical trials due to stringent treatment protocols.

## 9. Basic Medical Research Associated with BEV

### 9.1. Intranasal BEV Administration in Polymeric Nanoparticles

Recently, several interesting studies have been reported in the field of basic medical sciences. Using experimental animal models, intranasal BEV administration in polymeric nanoparticles was applied to improve the brain bioavailability of BEV [104]. The intranasal administration route is expected to directly reach the central nervous system by bypassing the blood–brain barrier through olfactory and trigeminal neural pathways, thus providing direct delivery to the brain [105]. Indeed, intranasally administered BEV-loaded poly (D,L-lactic-co-glycolic) acid (PLGA) nanoparticles exhibit a high anti-angiogenic effect [104]. PLGA was used to formulate nanoparticles for intranasal administration because of its biodegradability and biocompatibility and is already approved by the FDA for formulation development [106].

### 9.2. Targeting Autophagy

There is an emerging interest in targeting autophagy to enhance the therapeutic activity of BEV [107]. Autophagy plays pro-death and pro-survival roles in tumors, and there is increasing evidence that BEV induces autophagy of tumor cells to survive therapeutic stressors [108,109]. Hydroxychloroquine (HCQ) has been recently studied in many cancer types, especially as an adjuvant to increase the efficacy or limit drug resistance of other anti-cancer therapies because it inhibits lysosomal acidification and blocks autophagy [110]. Liu et al. [107] demonstrated that HCQ augments the therapeutic efficacy of BEV against GBM, and discovered that HCQ can sensitize GBM cells to BEV, which was partially dependent on autophagy inhibition.

### 9.3. Perivascular Microenvironment

The microenvironment of the perivascular space is emerging as a critical determinant of tumor cell growth and survival. It is well established that a population of cells with a cancer stem cell-like phenotype (e.g., expression of the CD133 cell surface marker and transcription factor Sox2) are resistant to radiation therapy [111]. Thus, alterations in the perivascular microenvironment may provide a niche that promotes and sustains the development of this cell population in various cancers, including GBM [112]. Furthermore, Müller-Greven et al. [112] reported that BEV is internalized by Sox2^+^/CD44^+^GBM tumor cells residing in the perivascular tumor niche.

### 9.4. Extracellular Vesicles in the Microenvironment

Intracellular communication of GBM cells with their direct microenvironment has also been studied [113]. Extracellular vesicles have been recently described as the main players in the GBM microenvironment, allowing tumor and stromal cells to exchange genetic and proteomic material [114]. Simon et al. [113] reported that treatment with BEV induces changes in the extracellular vesicle proteomic content that are associated with tumor progression and therapeutic resistance.

## 10. Conclusions

In this review, we discussed the recent clinical trials and findings on BEV use and provided suggestions for updating the BEV treatment regimen. Although BEV has a potential effect on the survival of patients with GBM, this effect relies on how BEV is used. To maximize the clinical benefits to patients, further efforts addressing the appropriate management of BEV treatment is required. However, data on the optimal management of BEV are still insufficient. We expect that the unique BEV approval in Japan, which allows for adaptive BEV use regardless of clinical stage, will contribute to the future accumulation of real-world clinical data.

## Figures and Tables

**Table 1:** Summary of studies on first-line BEV.

Author	Year	Study Category	Inclusion Criteria	Study Design	Number of Patients	Median PFS (Months)	Median OS (Months)
Chinot et al. [17]	2014	Phase III trial (AVAglio)	GBM, PS: 0–2	TMZ + BEV vs. TMZ	458 vs. 463	10.6 vs. 6.2 *	16.8 vs. 16.7
Gilbert et al. [18]	2014	Phase III trial (RTOG 0825)	GBM, KPS ≥ 70	TMZ + BEV vs. TMZ	312 vs. 309	10.7 vs. 7.3 *	15.7 vs. 16.1
Chinot et al. [21]	2016	Subanalysis (AVAglio)	GBM, PS: 0–2; without second-line treatment	TMZ + BEV vs. TMZ	120 vs. 105	8.4 vs. 4.8 *	11.6 vs. 8.0 *
Balana et al. [22]	2016	Phase II trial	GBM, PS: 0–2, PR/biopsy	TMZ + BEV vs. TMZ	48 vs. 45	4.8 vs. 2.2	10.6 vs. 7.7
Herrlinger et al. [26]	2016	Phase II trial (GLARIUS)	GBM, unmethylated *MGMT*, KPS ≥ 70	BEV + IRI vs. TMZ	116 vs. 54	9.7 vs. 6.0 *	16.6 vs. 17.5
Yonezawa et al. [27]	2016	Retrospective study	GBM, biopsy	TMZ + BEV vs. TMZ	20 vs. 11	NA	18.9 vs. 5.3 *
Hata et al. [28]	2017	Retrospective study	GBM, *IDH*-wildtype, PR/biopsy	TMZ + BEV vs. TMZ	13 vs. 19	NA	17.4 vs. 9.8
Wirsching et al. [25]	2018	Phase II trial (ARTE)	GBM, age ≥ 65	BEV vs. None	50 vs. 25	7.6 vs. 4.8 *	12.1 vs. 12.2
Hata et al. [29]	2020	Retrospective study	GBM, *IDH*-wildtype, unmethylated *MGMT*	TMZ + BEV vs. TMZ	26 vs. 24	NA	16.7 vs. 12.2 *

* indicates statistical significance. PFS, progression-free survival; OS, overall survival; GBM, glioblastoma; PS, performance status; TMZ, temozolomide; BEV, bevacizumab; KPS, Karnofsky performance status; IRI, irinotecan; PR, partial removal of tumors; NA, not available.

**Table 2:** Summary of studies on second or subsequent line BEV.

Author	Year	Study Category	Inclusion Criteria	Study Design	Number of Patients	Median PFS from PD (Months)	Median OS from PD (Months)
Taal et al. [42]	2014	Phase II trial (BELOB)	GBM, PS: 0–2; first recurrence	BEV + CCNU vs. BEV vs. CCNU	50 vs. 46 vs. 52	4 vs. 3 vs. 1	12 vs. 8 vs. 8
Field et al. [46]	2015	Phase II trial	GBM, PS: 0–2; first recurrence	BEV + CBDCA vs. BEV	60 vs. 62	3.5 vs. 3.5	6.9 vs. 7.5
Erdem-Eraslan et al. [43]	2016	Subanalysis (BELOB)	IGS-18 or classical GBM; first recurrence	BEV + CCNU vs. BEV vs. CCNU	43 vs. 35 vs. 37	4.2 vs. 2.9 vs. 1.4 *	11.9 vs. 8.3 vs. 7.9
Weathers et al. [47]	2016	Phase II trial	GBM, KPS ≥ 60; first recurrence	Low-dose BEV + CCNU vs. BEV	24 vs. 23	5.0 vs. 3.2	13.1 vs. 8.8
Badruddoja et al. [48]	2017	Phase II trial	GBM, KPS > 60; first recurrence	BEV + biweekly TMZ	30	5.3	11.7
Ajlan et al. [49]	2017	Retrospective study	GBM; recurrence (91%)	Low dose (<3 mg/kg/week) vs. high dose (≥3 mg/kg/week)	33 vs. 47	NA	39.0 vs. 17.3
Wick et al. [44]	2017	Phase III trial	GBM; first recurrence	BEV + CCNU vs. CCNU	288 vs. 149	4.2 vs. 1.5 *	9.1 vs. 8.6
Franceschi et al. [50]	2018	Retrospective study	GBM; second recurrence	BEV vs. chemotherapy	32 vs. 136	4.7 vs. 2.6 *	8.0 vs. 6.0 *
Brandes et al. [45]	2019	Phase II trial (TAMIGA)	GBM, PS: 0–2; first-line: TMZ + BEV	BEV + CCNU vs. CCNU	61 vs. 62	2.3 vs. 1.8	6.4 vs. 5.5

* indicates statistical significance. PD, progression disease; CCNU, lomustine; CBDCA, carboplatin.

**Table 3:** Summary of studies associated with relapse patterns.

Author	Year	Study Design	Relapse Pattern	Number of Patients	Median OS from PD (Months)	HR (95% CI)	*p* Value
Iwamoto et al. [66]	2009	BEV + multiple agents	Enhancing initial site	17 (46%)	4.5 (2.1–5.9)	Reference	
			New enhancing	6 (16%)		1.44 (0.51–4.08)	0.49
			Non-enhancing tumor	13 (35%)		2.25 (1.00–5.10)	0.05 *
Pope et al. [67]	2011	BEV alone	Local to local	37 (77%)	9.7 (8.1–11.8)	Reference	
			Local to diffuse	11 (23%)	9.2 (7.6–12.0)	1.6 (0.78–3.17)	NA
		BEV + IRI	Local to local	18 (45%)	11.5 (8.2–13.0)	Reference	
			Local to diffuse	22 (55%)	8.9 (7.8–11.0)	1.6 (0.83–3.16)	NA
Nowosielski et al. [68]	2014	BEV	T2 diffuse	15 (18%)	4.8 (1.8–7.7)	Reference	
			Flare-up of CE	35 (42%)	4.6 (3.4–5.7)	1.7 (1.3–2.2)	<0.001 *
			Primary non-responder	16 (19%)	3.0 (1.7–4.2)		
			T2 circumscribed	17 (21%)	1.6 (0.5–2.8)		
Kim et al. [69]	2015	BEV + multiple agents	Primary non-responder	21 (33%)	4.4 (2.9–5.9)	NA	<0.0001 *
			Flare-up of CE	25 (39%)	11.0 (7.7–14.3)		
			Non-enhancing infiltration	18 (28%)	9.0 (6.3–11.7)		
Bloch et al. [70]	2017	BEV + multiple agents	Nodular enhancement	33%	NA	NA	0.31
			Diffuse patchy enhancement	50%			
			Non-enhancing tumor	17%			

* indicates statistical significance. HR, hazard ratio; CI, confidence interval; CE, contrast enhancement.
